# Patient–reported fatigue prior to treatment is prognostic of survival in patients with acute myeloid leukemia

**DOI:** 10.18632/oncotarget.25787

**Published:** 2018-07-27

**Authors:** Tamara E. Lacourt, Annemieke Kavelaars, Maro Ohanian, Nina D. Shah, Samuel A. Shelburne, Andrew Futreal, Dimitrios P. Kontoyiannis, Cobi J. Heijnen

**Affiliations:** ^1^ Neuroimmunology Laboratory, Department of Symptom Research, The University of Texas MD Anderson Cancer Center, Houston, Texas, USA; ^2^ Department of Leukemia, The University of Texas MD Anderson Cancer Center, Houston, Texas, USA; ^3^ Department of Medicine, University of California San Francisco, San Francisco, California, USA; ^4^ Department of Infectious Disease, Infection Control, and Employee Health, The University of Texas MD Anderson Cancer Center, Houston, Texas, USA; ^5^ Department of Genomic Medicine, The University of Texas MD Anderson Cancer Center, Houston, Texas, USA

**Keywords:** fatigue, mortality, Kaplan-Meier, Cox regression, patient-reported outcome

## Abstract

Acute myeloid leukemia (AML) is associated with poor survival. While clinical prognostic factors of survival have been identified, the contribution of patient-reported symptoms has only received marginal attention. Fatigue is one of the most commonly reported symptoms of AML. There is some evidence that fatigue is associated with shorter survival in hematological malignancies. However, the prognostic effects of fatigue in a homogenous cohort of patients with *untreated* AML has not been tested. We here report results of a prospective study on the prognostic value of patient-reported fatigue prior to onset of treatment, for 2-year survival in 94 AML patients. Cox regression models controlling for demographic and clinical factors showed that those with severe fatigue (22%) had decreased survival rates (Hr = 2.255, 95% CI = 1.16-5.60, p = 0.019). Further exploration showed that fatigue was associated with increased plasma concentrations of IL-6 and TNF-α, but not with demographic or disease-related factors. In conclusion, we here show for the first time that the experience of severe fatigue *prior to remission induction chemotherapy* (IC) is prognostic for shorter survival in patients with AML of all ages. These findings point to the importance of interventions aimed at relieving fatigue especially before or in the early phases of treatment in order to improve survival.

## INTRODUCTION

Acute myeloid leukemia (AML), a hematological cancer in which cancerous myeloid blasts do not develop into mature white blood cells, is associated with poor survival [1]. While several prognostic clinical factors for survival have been identified, including cytogenetic abnormalities, performancestatus, co-morbidities, and age [2], evidence is emerging that patient-reported symptoms could add further precision to the prediction of survival [3, 4]. We here report on the prognostic value of fatigue, one of the most commonly reported symptoms of AML [5–7].

Fatigue is a highly prevalent symptom in patients with AML and is strongly associated with reduced quality of life [8]. Fatigue severity is at its peak prior to or at the start of treatment [8]. Indeed, over half of participants experience moderate to severe fatigue at this time [9, 10]. Fatigue has been associated with decreased survival in patients with other hematological malignancies (i.e., myelodysplastic syndrome, or MDS) [11, 12]. To the best of our knowledge, the prognostic effect of fatigue for survival in AML patients has been assessed in only two studies, with mixed results. Deschler et al. [13], followed a cohort of older patients (≥ 60 years) with either MDS or AML for up to 42 months and showed that higher fatigue at initiation of treatment was associated with shorter survival. As the study also included patients with a diagnosis of MDS, in whom fatigue has been shown to predict survival, it is unclear whether the results would be applicable to the AML patients. Furthermore, their age-restrictions limit the translation of the results to younger patients. In contrast, Timilshina et al. [14] studied AML patients only and did not find fatigue prior to or at the start of remission induction chemotherapy (IC) to be a prognostic factor in AML patients. However, their time frame of 60 days led to a very low mortality rate (i.e., 3.7%), which would have obscured any potential effects. Thus, it remains unclear whether fatigue assessed prior to initiation of treatment is prognostic of long-term survival in AML patients.

We here present on the predictive value of fatigue prior to remission induction chemotherapy (IC) on overall 2-year survival in patients with AML, controlling for common clinical and demographic prognostic factors. The exact mechanisms underlying AML-related fatigue are unclear and have not been addressed in prior survival studies. We here therefore report on associations of fatigue with clinical and treatment factors as well as biomarkers of inflammation.

## RESULTS

### Sample description

A total of 103 patients with AML were enrolled. One patient withdrew immediately after enrollment. Four patients were excluded based on medical history (end-stage renal failure; malignant pleural effusions secondary to acute megakaryotic leukemia; end-stage liver disease; progressing Alzheimer's disease). Another four patients were lost for follow up within one year of enrollment and excluded from analyses. Of the remaining 94 patients, baseline fatigue scores were available for 79 patients (84%). All baseline scores were obtained on the day of treatment onset or up to 5 days prior. Patients with available symptom data did not differ from patients with missing symptom data in age, clinical laboratory values, performance status, AML type, or cytogenetic risk (all: p > 0.05). Furthermore, missingness was not associated with survival (p = 0.29). However, patients with missing symptom data more often had a previous diagnosis of a hematological malignancy, as compared to patients with available symptom data (40% vs. 8%, p = 0.001). Blood plasma samples were available for a subset of 55 patients. Fatigue scores did not differ between patients with available blood plasma samples and those without (p = 0.10). Details on the sample with baseline fatigue are provided in Table 1. Forty-six patients (58%) reported low fatigue, 16 (20%) moderate fatigue, and 17 (22%) severe fatigue.

**Table 1:** Patient characteristics (n = 79) split by fatigue severity

	Low fatigue (n=46)	Moderate fatigue (n=16)	High fatigue (n=17)
Age	56.09 (4.46; 18-84)	46.31 (3.96; 22-67)	57.77 (3.21; 26-77)
Sex			
Male	26 (56)	6 (38)	9 (53)
Female	20 (44)	10 (62)	8 (47)
Previous hematologic cancer			
Yes	4 (9)	0 (0)	2 (12)
No	42 (91)	16 (100)	15 (88)
Charlson Comorbidity Index			
0	38 (83)	15 (94)	10 (59)
1	7 (15)	0 (0)	6 (35)
2+	1 (2)	1 (6)	1 (6)
ECOG performance status			
0	10 (22)	5 (31)	3 (18)
1	35 (76)	8 (50)	7 (41)
2	1 (2)	3 (19)	7 (41)
AML type			
De novo	41 (89)	15 (94)	15 (88)
Treatment-related	5 (11)	1 (6)	2 (12)
Cytogenetic Risk Group			
Favorable	6 (13)	2 (12)	3 (18)
Intermediate/diploid	32 (70)	14 (88)	8 (47)
Unfavorable	8 (17)	0 (0)	6 (35)
Induction treatment			
High intensity^1^	30 (65)	13 (81)	12 (71)
Hypomethylators	14 (31)	2 (13)	3 (17)
Low intensity^2^	2 (4)	1 (6)	2 (12)
Received transplant after IC	10 (22)	11 (69)	6 (35)
*Clinical laboratory values*			
Hb (g/dL)	9.84 (0.35; 7.9-23.0)	9.66 (0.22; 8.0-11.3)	9.32 (0.23; 7.8-12.2)
Albumin (g/dL)	3.35 (0.07; 2.7-4.7)	3.42 (0.13; 2.3-4.1)	3.30 (0.11; 2.3-4.1)
Fatigue	1.80 (0.20; 0-4)	5.25 (0.11; 5-6)	8.18 (0.32; 7-10)

^1^ cytotoxic agents ±fludarabine; ^2^ cytarabine and cladribine only (n=4) or cytarabine with omacetaxine (n=1).

Abbreviations: M, mean; SEM, standard error of the mean; ECOG, Eastern Cooperative Oncology Group; AML, acute myeloid leukemia; IC, induction chemotherapy; Hb, hemoglobin;

Figures represent M (SEM; range) or no. of patients (%).

### Prognostic value of fatigue for survival

Thirty-five patients (44%) died in the 2-year interval and nine patients were lost to follow-up between 12 and 24 months since onset of IC. The following demographic and disease-related factors were also associated with decreased survival: an older age; ECOG of 2; treatment-related etiology of AML; previous diagnosis of a hematological cancer; high cytogenetic risk (Table 2). Sex and presence of comorbid conditions were not associated with survival.

**Table 2:** Univariate and multivariate cox regression models for overall survival

	Univariate models			Multivariate model		
	HR	95% CI	P	HR	95% CI	P
Age	1.030	1.007-1.054	**0.009**	1.019	0.992-1.047	0.17
Sex^1^	0.801	0.428-1.500	0.49	N/A		
ECOG performance status^2^	2.798	1.095-7.148	**0.032**	1.252	0.397-3.942	0.70
Etiology^3^	2.758	1.268-5.999	**0.011**	0.944	0.358-2.491	0.91
Previous hematologic cancer^4^	5.473	2.554-11.729	**<0.001**	5.266	1.827-15.180	**0.002**
Cytogenetic risk^5^						
Intermediate risk	1.387	0.418-4.607	0.59	1.468	0.421-5.120	0.55
High risk	5.650	1.603-19.914	**0.007**	4.735	1.197-18.730	**0.027**
CCI^6^						
1	1.401	0.616-3.183	0.42	N/A		
≥2	1.727	0.413-7.222	0.45	N/A		
Fatigue^7^						
Moderate severity	0.533	0.182-1.560	0.25	1.057	0.336-3.330	0.92
High severity	2.122	1.012-4.451	**0.047**	2.554	1.164-5.603	**0.019**

Reference:^1^ male; ^2^ ECOG = 0; ^3^ de novo; ^4^ no previous hematological malignancy; ^5^ favorable cytogenetics; ^6^ CCI-score =0; ^7^ low fatigue. Abbreviations: HR, hazard ratio; CI, confidence interval; ECOG, Eastern Cooperative Oncology Group; CCI, Charlson Comorbidity Index.

**Figure 1:**
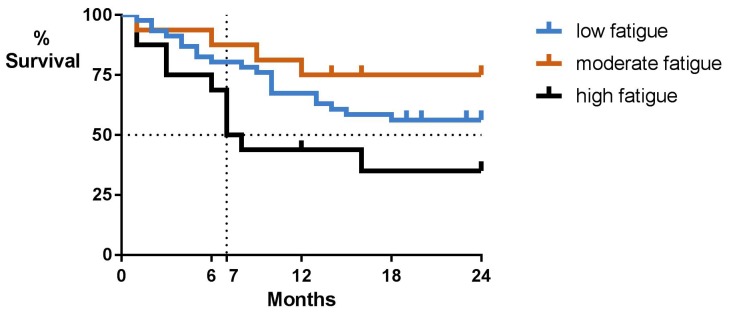
Overall survival by baseline patient-reported fatigue severity Low fatigue indicates a fatigue severity score ≤ 4, moderate fatigue a score of 5-6, and high fatigue a score ≥7 on a 0-10 scale.

Univariate Cox regression models yielded significant prognostic effects of fatigue on survival. Severe, but not moderate fatigue, was related to decreased overall survival as contrasted to low fatigue. A multivariate model including the identified clinical and demographic factors confirmed that high fatigue severity was associated with decreased overall survival. At the 2-year follow-up, 56% of patients with low fatigue were alive, 75% of patients with moderate fatigue and 35% of patients with high fatigue. Median survival in the group with high fatigue was 7 months (Figure 1). While Figure 1 suggests that moderate fatigue might be associated with improved survival, this effect was not significant (Table 2).

An explorative model showed that fatigue was not prognostic of remission rate (HRmoderate fatigue = 1.025, p = 0.93; HRhigh fatigue = 1.164, p = 0.64).

### Associations of fatigue

**Figure 2:**
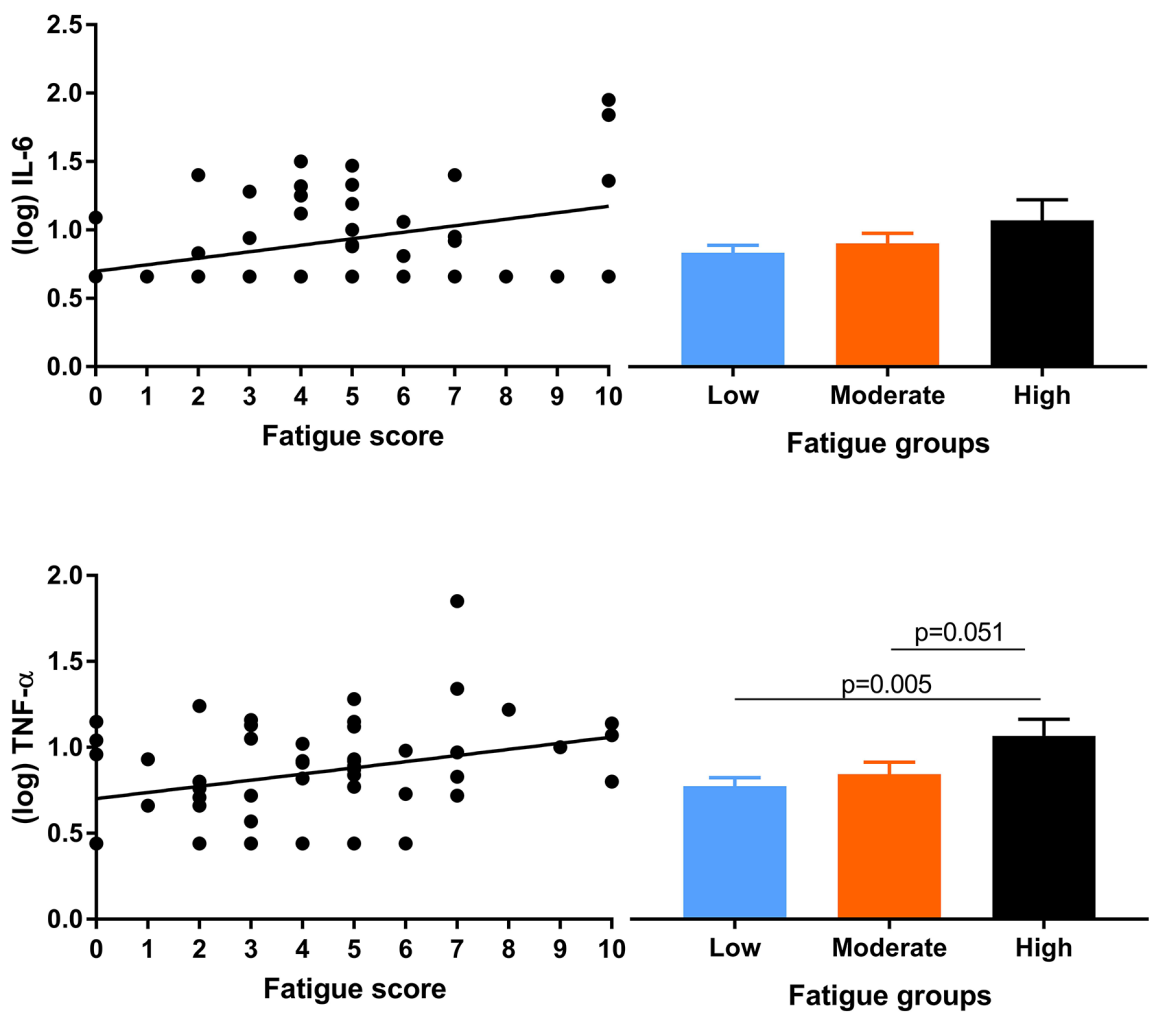
Scatterplots for associations of fatigue with IL-6 and TNF-α (left panels) and mean (log transformed) cytokine concentrations per fatigue group (right panels)

Fatigue was not associated with age or gender. No significant associations were observed with disease-related factors, nor with albumin, hemoglobin or with WBC (Table 3). Fatigue was associated with higher levels of TNF-α and IL-6 but not with any of the other markers (Table 3 and Figure 2). Group comparisons showed significantly higher TNF-α concentrations in the group with high fatigue (p = 0.017). Differences in IL-6 were not significant (p = 0.34).

**Table 3:** Associations between fatigue and demographic and disease-related factors, clinical laboratory values, and plasma concentrations of inflammatory biomarkers

	Fatigue	
	statistic	p-value
Age	r = -0.05	0.67
Gender	t = -0.84	0.41
Cytogenetic risk	F = 2.092	0.13
ECOG	t = 0.026	0.98
Etiology	t = 0.382	0.70
Previous hematologic cancer	t = -0.55	0.59
CCI	F = 1.840	0.17
Albumin	r = 0.01	0.94
Hemoglobin	r = -0.04	0.70
WBC	ρ = -0.03	0.81
IL-6^1^ (n=53)	r = 0.40	0.003
TNFa^1^ (n=53)	r = 0.34	0.012
IL-1ra^1^ (n=51)	r = 0.11	0.47
TNFRI^1^ (n=51)	r = 0.10	0.50

^1^ log transformed.

## DISCUSSION

We here show for the first time that the experience of severe fatigue *prior to remission induction chemotherapy* (IC) might be prognostic for shorter overall survival in patients with AML of all ages. This effect was found above and beyond the known clinical prognostic factors such as age, performance status, and cytogenetic risk. Fatigue was not prognostic of remission rate, suggesting that the effects on survival are not through a reduced responsivity to the treatment. The association between fatigue and survival has previously been reported for patients with MDS who were tested for fatigue within six months of diagnosis [11] as well as in an elderly population including both AML and MDS patients [13]. We here extend these earlier findings with a single fatigue-item assessed prior to treatment in a sample of AML patients with a wide age-range. With approximately one-fifth of our sample experiencing severe fatigue prior to IC, it is clear that this symptom represents a significant clinical problem. While moderate fatigue was not found to be prognostic for shorter survival, the univariate survival curves suggest that moderate fatigue might have a protective effect, which warrants further study.

While the mechanisms underlying cancer-related fatigue are still unclear, previous reports point to associations with psychological distress, increased inflammation, and, in some cases, decreased hemoglobin and albumin levels [15]. The association with hemoglobin and albumin levels was not apparent in our study cohort. However, we did observe the previously reported association between fatigue and the inflammatory biomarker TNF-α [9, 10]. As no associations were found for the other assessed inflammation biomarkers and the associations with TNF-α and IL-6 are of moderate magnitude, we conclude that inflammation is not the only driver of fatigue. More recently, we proposed that fatigue in cancer patients may be a reflection of decreased mitochondrial function as a result of the cumulative effects of the cancer, inflammatory processes, and distress [16, 17]. Currently, preliminary evidence for this hypothesis comes mainly from populations with solid tumors (e.g., breast cancer). However, it is interesting to note that leukemic cells produce higher levels of reactive oxidative species (ROS) compared to normal leukocytes [18]. High levels of ROS lead to oxidative stress, which is known to damage mitochondria. Whether mitochondrial function in normal leukocytes is reduced in fatigued patients with AML needs to be determined.

Some limitations warrant mentioning. As the here reported analyses were part of retrospectively formulated research objectives, not all utilized eligibility criteria were relevant for the here presented analyses and might have biased the results. Specifically, patients with poor performance status were excluded from the study, while those patients potentially would have high fatigue and poorer survival chances. Further, the criteria resulted in a sample heterogeneous for disease etiology and past diagnoses of hematological malignancies. In addition, the cohort size is relatively modest for survival analyses and inflammatory biomarker information was available for only a subset of patients. Replication of our findings in larger samples screened according to more appropriate eligibility criteria is therefore necessary. However, our finding on the prognostic effects of fatigue match findings in other hematological cancers, underlining the validity of our results. Another potential limitation of the current study was the use of a single item to assess fatigue, rather than extensive validated questionnaires to assess the full scope of this experience. Use of more extensive measures of fatigue might improve our understanding of how fatigue predicts survival. Nevertheless, the fatigue item used in the current study has been validated and has great clinical relevance, as single-item surveys are easily implemented in the clinic and do not add additional burden to the already taxed patient. This is especially important as patients with AML generally are hospitalized for treatment very soon after diagnosis and are often overwhelmed with the implications of the diagnosis and the major changes in their daily life this ensues.

In conclusion, we have shown here that severe fatigue experienced before onset of IC is predictive of poor survival, above and beyond the demographic and disease-related variables known to be associated with survival. These findings are important because they allow for a better prediction of survival and pose promising avenues for interventions aimed at relieving fatigue. The observation that fatigue might predict relapse after initial remission suggests that patients who initially present with high fatigue would benefit from increased surveillance after remission is obtained as well as stronger maintenance therapies to prevent recurrence.

## MATERIALS AND METHODS

### Design

Data for this study was collected prospectively for study objectives reported elsewhere [19, 20]. The here reported survival analyses were part of retrospectively formulated secondary study objectives. Primary goals of the prospective study aimed at assessing patient-reported symptoms before and during IC and studying the role of gastrointestinal microbiome in infectious complications during IC (see [19] and [20] for prior reports). Patients with either AML or MDS were recruited shortly after diagnosis. Assessments, including patient-reported outcomes, and collection of a blood and stool sample, were done at baseline (prior to or within 8 hours after start of IC) and during IC until neutrophil count recovery (an absolute neutrophil count > 500 cells/μL), which generally occurred in the third or fourth week of IC.

We here report on baseline symptom report of patients with AML, which made up the majority of the study sample (99%). The study (protocol PA13-0339) was approved by The University of Texas MD Anderson Cancer Center Internal Review Board and was conducted in compliance with the Declaration of Helsinki. All participants provided written informed consent.

### Study population

Adult patients (≥ 18 years of age) were considered eligible when presenting at the Department of Leukemia at The University of Texas MD Anderson Cancer Center in Houston, Texas, between September 2013 and August 2015 with a confirmed diagnosis of AML, and admitted or planned to be admitted to receive IC within two weeks. Patients were required to have an Eastern Cooperative Oncology Group (ECOG) performance status of 0–2, a calcium phosphorus product ≤ 4.0 mmol^2^/L^2^ (50 mg^2^/dL^2^), and adequate organ function. Patients were excluded if they had another concurrent malignancy or had received any chemotherapeutic or investigational anticancer drug within the previous two weeks, had any cardiac risk factor, or had any unresolved grade 1+ toxicity from previous therapy. Patients with an active bacterial infection at baseline - defined as current receipt of therapeutic (not prophylactic) antibiotics- were also excluded for the purpose of elsewhere reported study aims.

### Fatigue assessment

Patient-reported fatigue was assessed with the fatigue-item from the MD Anderson Symptom Inventory (MDASI) [21]. The MDASI is a 13-item scale asking for the severity of 11 somatic and 2 psychological symptoms at their worst in the last 24 hours. Patients rate their severity on scale of 0 (“not present”) to 10 (“As bad as you can imagine”). The fatigue item of the MDASI asked to rate “Your fatigue (tiredness) at its WORST”. This item is identical to the item of the Brief Fatigue Inventory (BFI), which is commonly used as indicator of cancer-related fatigue e.g., [15]. In accordance with MDASI and BFI guidelines [22], patients were categorized into low (0-4), moderate (5-6) or severe fatigue (score ≥7).

### Demographic and clinical laboratory factors

Demographic and clinical (laboratory) information as well as cytogenetic risk were obtained from electronic medical records. Clinical laboratory values were obtained for or as close as possible to the date of the MDASI assessment. Cytogenetic risk was determined according to the National Comprehensive Cancer Network Guidelines for AML. Information regarding treatment for AML within the 2-year time span was also obtained from the medical records.

### Treatment response and survival

We obtained remission status and time of remission from the medical records. Patients were considered in remission when their bone marrow blast percentage was below 5% and neutrophil count had recovered.

Overall survival was assessed from the onset of IC to the date of death due to any cause within a two-year time span from the onset of IC. Date and cause of death was obtained from the medical records.

### Biomarkers of inflammation

Processing of blood samples and determination of plasma concentrations is described elsewhere [20]. In short, peripheral blood samples collected in EDTA-anticoagulating tubes were spun down at 3000 rpm for 10 minutes immediately after collection after which plasma was stored at –80°C until batchwise analysis. An array of cytokines, adhesion molecules, and damage-associated molecular patterns was assessed, which are reported elsewhere. We here report only on plasma concentrations of the pro-inflammatory cytokines and receptors commonly reported to be associated with fatigue i.e., interleukin (IL)-6, IL-1 receptor antagonist (IL-1Ra), tumor necrosis factor (TNF)-α, and soluble TNF receptor I (sTNF-RI)), all of which were assessed with Magnetic Luminex Screening Assays (R&D Systems, Minneapolis, MN). Cytokine concentrations were log-transformed to obtain normal distributions.

### Statistical analyses

Patients who were lost to follow-up within one year of IC onset were excluded from analyses. Patients who were alive at the two-year follow up were censored at the date of the last recorded contact with the clinic.

The prognostic value of fatigue was assessed in multivariate Cox Regression model including demographic and clinical covariates. Covariates were pre-identified in univariate Cox Regression models including age, sex, comorbidity (Charlson Comorbidity Index), AML etiology (de-novo or related to prior cancer treatment), ECOG performance status (0 vs. 1-2), previous diagnoses of hematological cancers including AML, and cytogenetic risk. Those risk factors identified through univariate analyses (i.e., showing an association at p < 0.05) were subsequently included in the final multivariate model.

Associations between fatigue and other (continuous) variables were computed with Pearson's r and Spearman's rho statistics, the latter for WBC, which had a non-normal distribution. Associations with nominal variables were assessed with student's *t*-tests and univariate analyses of variance. All analyses were performed using IBM SPSS Statistics Version 24 with alpha set at 0.05.

## Abbreviations

AML: Acute myeloid leukemia
MDS: myeloid dysplastic syndrome
IC: remission induction chemotherapy
IL: interleukin
TNF: tumor necrosis factor
WBC: white blood cell
MDASI: MD Anderson Symptom Inventory
BFI: Brief Fatigue Inventory

## References

[1] https://www.cancer.org (2017). Cancer Facts & Figures 2017.

[2] De Kouchkovsky I, Abdul-Hay M (2016). ‘Acute myeloid leukemia: a comprehensive review and 2016 update’. Blood Cancer J.

[3] Oliva EN, Nobile F, Alimena G, Ronco F, Specchia G, Impera S, Breccia M, Vincelli I, Carmosino I, Guglielmo P, Pastore D, Alati C, Latagliata R (2011). Quality of life in elderly patients with acute myeloid leukemia: patients may be more accurate than physicians. Haematologica.

[4] Akaho R, Sasaki T, Mori S, Akiyama H, Yoshino M, Hagiya K, Nakagome K, Sakamaki H (2003). Psychological factors and survival after bone marrow transplantation in patients with leukemia. Psychiatry Clin Neurosci.

[5] Bryant AL, Walton AL, Shaw-Kokot J, Mayer DK, Reeve BB (2015). Patient-reported symptoms and quality of life in adults with acute leukemia: A systematic review. Oncol Nurs Forum.

[6] Albrecht TA (2014). Physiologic and psychological symptoms experienced by adults with acute leukemia: An integrative literature review. Oncol Nurs Forum.

[7] Zordan R, Manitta V, Nandurkar H, Cole-Sinclair M, Philip J (2014). Prevalence and predictors of fatigue in haemo-oncological patients. Intern Med J.

[8] Schumacher A, Wewers D, Heinecke A, Sauerland C, Koch OM, Van De Loo J, Büchner T, Berdel WE (2002). Fatigue as an important aspect of quality of life in patients with acute myeloid leukemia. Leuk Res.

[9] Fung FY, Li M, Breunis H, Timilshina N, Minden MD, Alibhai SM (2013). Correlation between cytokine levels and changes in fatigue and quality of life in patients with acute myeloid leukemia. Leuk Res.

[10] Meyers CA, Albitar M, Estey E (2005). Cognitive impairment, fatigue, and cytokine levels in patients with acute myelogenous leukemia or myelodysplastic syndrome. Cancer.

[11] Efficace F, Gaidano G, Breccia M, Voso MT, Cottone F, Angelucci E, Caocci G, Stauder R, Selleslag D, Sprangers M, Platzbecker U, Ricco A, Sanpaolo G (2015). Prognostic value of self-reported fatigue on overall survival in patients with myelodysplastic syndromes: a multicentre, prospective, observational, cohort study. Lancet Oncol.

[12] Buckstein R, Wells RA, Zhu N, Leitch HA, Nevill TJ, Yee KW, Leber B, Sabloff M, St Hilaire E, Kumar R, Geddes M, Shamy A, Storring J (2016). Patient-related factors independently impact overall survival in patients with myelodysplastic syndromes: an MDS-CAN prospective study. Br J Haematol.

[13] Deschler B, Ihorst G, Platzbecker U, Germing U, Marz E, de Figuerido M, Fritzsche K, Haas P, Salih HR, Giagounidis A, Selleslag D, Labar B, de Witte T (2013). Parameters detected by geriatric and quality of life assessment in 195 older patients with myelodysplastic syndromes and acute myeloid leukemia are highly predictive for outcome. Haematologica.

[14] Timilshina N, Breunis H, Brandwein JM, Minden MD, Gupta V, O’Neill S, Tomlinson GA, Buckstein R, Li M, Alibhai SM (2014). Do quality of life or physical function at diagnosis predict short-term outcomes during intensive chemotherapy in AML?. Ann Oncol.

[15] Wang XS, Giralt SA, Mendoza TR, Engstrom MC, Johnson BA, Peterson N, Broemeling LD, Cleeland CS (2002). Clinical factors associated with cancer-related fatigue in patients being treated for leukemia and non-Hodgkin's lymphoma. J Clin Oncol.

[16] Vichaya EG, Chiu GS, Krukowski K, Lacourt TE, Kavelaars A, Dantzer R, Heijnen CJ, Walker AK (2015). Mechanisms of chemotherapy-induced behavioral toxicities. Front Neurosci.

[17] Lacourt TE, Heijnen CJ (2017). Mechanisms of Neurotoxic Symptoms as a Result of Breast Cancer and Its Treatment: Considerations on the Contribution of Stress, Inflammation, and Cellular Bioenergetics. Curr Breast Cancer Rep.

[18] Zhou F, Shen Q, Claret FX (2013). Novel roles of reactive oxygen species in the pathogenesis of acute myeloid leukemia. J Leukoc Biol.

[19] Galloway-Pena JR, Smith DP, Sahasrabhojane P, Ajami NJ, Wadsworth WD, Daver NG, Chemaly RF, Marsh L, Ghantoji SS, Pemmaraju N, Garcia-Manero G, Rezvani K, Alousi AM (2016). The role of the gastrointestinal microbiome in infectious complications during induction chemotherapy for acute myeloid leukemia. Cancer.

[20] Lacourt TE, Kavelaars A, Galloway-Pena JR, Sahasrabhojane PV, Shah ND, Futreal A, Kontoyiannis DP, Shelburne SA, Heijnen CJ (2018). Associations of inflammation with symptom burden in patients with acute myeloid leukemia. Psychoneuroendocrinology.

[21] Cleeland CS, Mendoza TR, Wang XS, Chou C, Harle MT, Morrissey M, Engstrom MC (2000). Assessing symptom distress in cancer patients: The M. D. Anderson Symptom Inventory. Cancer.

[22] Cleeland C (2016). The M.D. Anderson Symptom Inventory User Guide. Version 1.

